# Inflammatory cytokines, T lymphocyte subsets, and ritonavir involved in liver injury of COVID-19 patients

**DOI:** 10.1038/s41392-020-00363-9

**Published:** 2020-10-31

**Authors:** Shengtao Liao, Ke Zhan, Li Gan, Yang Bai, Jinfang Li, Guodan Yuan, Ying Cai, An Zhang, Song He, Zhechuan Mei

**Affiliations:** 1 Department of Gastroenterology, The Second Affiliated Hospital of Chongqing Medical University, 400010 Chongqing, China; 2 Laboratory of Forensic Medicine and Biomedical Informatics, College of Basic Medicine, Chongqing Medical University, 400016 Chongqing, China; 3 Department of Respiratory and Critical Care Medicine, The First Affiliated Hospital of Chongqing Medical University, 400010 Chongqing, China; 4 Department of Neurology, The Second Affiliated Hospital of Chongqing Medical University, 400010 Chongqing, China; 5 Department of Critical Care Medicine, Chongqing Public Health Medical Center, 400036 Chongqing, China; 6 Department of Critical Care Medicine, The Second Affiliated Hospital of Chongqing Medical University, 400010 Chongqing, China

**Keywords:** Infectious diseases, Infectious diseases

**Dear Editor**,

Coronavirus disease 2019 (COVID-19) is a type of novel coronavirus and no specific treatment is currently available. Apart from damages to the lung, COVID-19 is also able to trigger liver injury. Numerous observational studies have revealed that elevation of liver enzymes, including alanine aminotransferase (ALT) and aspartate aminotransferase (AST), was detected in some COVID-19 patients, especially in severe cases.^1^ Unfortunately, the underlying mechanisms and risk factors of COVID-19-induced liver damage have not been completely elucidated. Therefore, identification of novel risk factors in liver injury of COVID-19 patients is essential for the prevention and treatment of liver damage. In this study, 192 patients with COVID-19 hospitalized in Chongqing Public Health Center were recruited to identify the risk factors in COVID-19 patients with liver injury.

The characteristics of 163 mild and 29 severe patients with COVID-19 were analyzed in this study. The results indicated that age and hospitalization time of severe patients were significantly higher compared to mild cases (*P* < 0.05, Supplementary Table S1). Additionally, the respiratory (fever, shortness of breath, and chest tightness) and gastrointestinal symptoms (anorexia) in severe patients were more obvious (*P* < 0.05, Supplementary Table S1). Furthermore, liver injury was more commonly detected in the severe group, in whom the expression levels of inflammatory cytokines (procalcitonin, C-reactive protein, erythrocyte sedimentation rate, interleukin (IL)-6, IL-10, and IL-17A) were remarkably elevated (*P* < 0.05, Supplementary Table S1). The numbers of T lymphocyte subsets (CD3^+^, CD4^+^ and CD8^+^ T cells) in patients with severe COVID-19 were notably decreased compared to mild patients (*P* < 0.05, Supplementary Table S1).

Further studies on liver function alterations in patients with mild and severe COVID-19 suggested that the levels of albumin were significantly reduced, and the production of ALT, AST, and lactate dehydrogenase (LDH) was remarkably elevated in patients with severe COVID-19 at admission. Furthermore, the severity of liver injury in patients was aggravated during hospitalization, and the levels of ALT, AST, alkaline phosphatase and LDH in severe patients with COVID-19 were significantly increased compared to the mild group (Supplementary Fig. S2). When the patients tested negative for COVID-19 and reached the discharge standard, liver function of both the experimental groups was significantly improved. These findings suggested that, during the development of COVID-19, liver injury could be aggravated. Taken all together, age, respiratory and gastrointestinal symptoms, the levels of inflammatory cytokines, numbers of T lymphocyte subsets, and degree of liver injury were associated with COVID-19 severity.

To further analyze the risk factors in liver injury of COVID-19 patients, 75 patients (39.06%) with liver injury and 117 patients without liver injury were recruited at admission. More male participants were found in the liver injury group compared to patients without liver injury (*P* < 0.05, Supplementary Table S2). In addition, more obvious gastrointestinal symptoms (anorexia), reduced number of T lymphocyte subsets (CD3^+^ and CD4^+^ T cells), and upregulation of inflammatory cytokines (r-interferon, tumor necrosis factor (TNF)-α, IL-2, IL-4, IL-10, and IL-17A) were detected in patients with severe COVID-19. Logistic regression was performed to identify potential risk factors in COVID-19 patients with liver injury at admission. Univariate analysis revealed that gender, TNF-α, IL-2, IL-4, IL-17A, CD3^+^ T, and CD4^+^ T cells were novel risk factors in COVID-19 patients with liver injury (*P* < 0.05,Supplementary Table S3). Multivariate analysis indicated that gender (odds ratio (OR) = 0.31, 95% confidence interval (CI) = 0.11–0.80, *P* = 0.007), IL-2 (OR = 4.07, 95% CI = 1.25–14.20, *P* < 0.001), IL-17A (OR = 1.63, 95% CI = 1.23–2.40, *P* = 0.004), and CD4^+^ T (OR = 3.90, 95% CI = 1.41–12.17, *P* < 0.001) cells were independent risk factors in COVID-19 patients with liver injury (*P* < 0.05, Supplementary Table S3).

During hospitalization, the number of patients with liver injury was increased significantly, as liver injury was detected in 133 COVID-19 patients (69.27%), while 59 patients exhibited no liver damage. Interestingly, liver injury was detected in 25 out of 29 (86.21%) severe COVID-19 patients. The results suggested that the proportion of male patients, nausea, and ritonavir/antibiotics treatment were significantly higher in participants with liver injury (*P* < 0.05, Supplementary Table S4). In addition, patients with liver injury exhibited reduced number of T lymphocyte subsets (CD3^+^, CD4^+^ and CD8^+^ T cells), and increased levels of inflammatory cytokines (TNF-α, IL-2, IL-6, and IL-10) during hospitalization (*P* < 0.05, Supplementary Table S4). Logistic regression was carried out to identify putative risk factors in COVID-19 patients with liver injury during hospitalization. Univariate analysis revealed that ritonavir, IL-2, IL-6, IL-10, CD4^+^ T, and CD8^+^ T cells were novel risk factors of COVID-19 patients with liver injury during hospitalization (*P* < 0.05, Table 1). Multivariate analysis suggested that ritonavir (OR = 4.75, 95% CI = 1.89–16.55, *P* < 0.001), IL-6 (OR = 3.27, 95% CI = 2.09–5.60, *P* < 0.001), CD4^+^ (OR = 0.99, 95% CI = 0.95–1.23, *P* = 0.010) and CD8^+^ T cells (OR = 1.38, 95% CI = 0.97–1.96, *P* < 0.001) were independent risk factors in COVID-19 patients with liver injury (*P* < 0.05, Table 1).

**Table 1:** Univariate and multivariate analyses of potential risk factors in COVID-19 patients with liver injury during hospitalization

Variable	Univariable OR (95% CI)	*P* value	Multivariable OR (95% CI)	*P* value
Sex	0.46 (0.17–1.2)	0.117		
Nausea	2.21 (0.79–7.19)	0.152		
Platelet count, ×10^9^/L	1 (1–1.01)	0.670		
Hemoglobin, g/L	1.02 (1–1.05)	0.127		
Monocyte count, ×10^9^/L	3.86 (0.67–49.3)	0.247		
Ritonavir	4.15 (2.11–15.83)	<0.001	4.75 (1.89–16.55)	<0.001
Antibiotics	2.7 (80.86–12.47)	0.122		
CD4^+^ T cell, per μL	0.92 (0.86–0.97)	0.018	0.99 (0.95–1.23)	0.010
CD8^+^ T cell, per μL	1.43 (1.01–2.4)	0.007	1.38 (0.97–1.96)	<0.001
CD4^+^ T/CD8^+^ T cell	1.08 (1.03–1.15)	0.057		
CD3^+^ T cell, per μL	0.41 (0.22–0.74)	0.052		
TNF-α, pg/mL	1.53 (1.05–2.49)	0.051		
IL-10, pg/mL	1.78 (1.34–2.51)	0.001		
IL-6, pg/mL	2.63 (1.18–7.66)	<0.001	3.27 (2.09–5.60)	<0.001
IL-2, pg/mL	1.33 (1.15–1.62)	0.001		

Dysregulation of immune response was observed in COVID-19 patients.^2^ During the development of COVID-19, the expression of programmed cell death receptor 1 and Tim-3 in T lymphocytes was increased, suggesting the depletion of T cells.^3^ In consistence with these findings, the results of this study suggested that the numbers of T lymphocyte subsets (CD3^+^, CD4^+^ and CD8^+^ T cells) were significantly reduced in patients with liver injury during hospitalization. In addition, CD4^+^ T cells were an independent risk factor in COVID-19 patients with liver injury at admission. Furthermore, CD4^+^ and CD8^+^ T cells were potential independent risk factors in COVID-19 patients with liver injury during hospitalization. Taken all together, depletion of T lymphocytes was detected to serve essential roles on liver injury in COVID-19 patients.

Severe acute respiratory syndrome coronavirus 2 (SARS-CoV-2)-mediated production of inflammatory cytokines could contribute to liver injury.^4^ In this study, the expression levels of r-interferon, TNF-α, IL-2, IL-4, IL-10, and IL-17A were remarkably elevated in patients with liver injury at admission. Multivariate analysis indicated that IL-2 and IL-17A were independent risk factors in COVID-19 patients with liver injury at admission, suggesting IL-2 and IL-17A were key inflammatory cytokines that could lead to liver injury. However, the inflammatory factors continued to change during the development of COVID-19. The results of this study revealed that COVID-19 patients with liver injury exhibited increased levels of inflammatory cytokines (TNF-α, IL-2, IL-6, and IL-10) compared to participants without liver injury. Furthermore, multivariate analysis revealed that IL-6 was an independent risk factor in COVID-19 patients with liver injury during hospitalization. In summary, an increased production of inflammatory cytokines was detected in COVID-19 patients, which was associated with liver injury. In clinical practice, alteration of inflammatory cytokines should be closely monitored during the treatment.

At present, no specific treatment is available for COVID-19 patients. The commonly used antiviral drugs lopinavir/ritonavir are mainly metabolized in the liver, and the side effects include liver dysfunction. Previous studies have suggested that use of lopinavir/ritonavir was associated with significantly aggravated liver injury.^5^ In this study, the proportion of ritonavir- and antibiotics-treated cases was significantly increased in COVID-19 patients with liver injury. Multivariate analysis indicated that ritonavir treatment was an independent risk factor in COVID-19 patients with liver injury.

In conclusion, liver injury was found in many COVID-19 patients, especially those with severe symptoms. The underlying mechanisms of liver injury in COVID-19 patients mainly involve SARS-CoV-2-mediated liver damage, immune response, and cytokine production, as well as drug-induced liver injury. Therefore, during the treatment of COVID-19, intervention targeting CD4^+^ and CD8^+^ T cells, antagonist of inflammatory cytokines, could be a promising therapeutic strategy for the treatment of liver injury in COVID-19 patients, and drug-induced liver damage should also be avoided.

## Supplementary information

Inflammatory cytokines, T lymphocyte subsets and ritonavir involved in liver injury of COVID-19 patients
