# Supplementary material for: Inflammatory cytokines, T lymphocyte subsets, and ritonavir involved in liver injury of COVID-19 patients
Source: Signal Transduct Target Ther. 2020 Oct 31;5:255. doi: 10.1038/s41392-020-00363-9 (PMC7599975; doi:10.1038/s41392-020-00363-9)
Supplement: Supplementary file 1 — Inflammatory cytokines, T lymphocyte subsets and ritonavir involved in liver injury of COVID-19 patients [file 41392_2020_363_MOESM1_ESM.docx]

**Supplemental Material for**

**Inflammatory cytokines, T lymphocyte subsets and ritonavir involved in liver injury of COVID-19 patients**

Shengtao Liao^1*^, Ke Zhan^1*^, Li Gan^2*^, Yang Bai^3*^, Jinfang Li^4^, Guodan Yuan^5^, Ying Cai^6^, An Zhang^6✉️^, Song He^1✉️^, Zhechuan Mei^1✉️^.

^✉️^**Correspondence to:**

Zhechuan Mei ([meizhechuan@cqmu.edu.cn](mailto:meizhechuan@cqmu.edu.cn)), Song He (hedoctor65@cqmu.edu.cn), An Zhang (zhangan@hospital.cqmu.edu.cn);

^*^These authors contributed equally: Shengtao Liao, Ke Zhan, Li Gan and Yang Bai;

**This PDF file includes:**

Materials and Methods

Figures S1 to S2

Tables S1 to S4

**Materials and methods**

**Clinical samples**

In our research, a single-center retrospective study was carried out in consecutive inpatient at Chongqing Public Health Center, Chongqing, China, from January to March 2020. A total of 208 patients diagnosed with COVID-19 were enrolled. The clinical and laboratory data of COVID-19 patients were collected and analyzed. The study protocol and informed consent form were reviewed and approved by the Ethics Review Committee of Chongqing Public Health Center(2020-025-KY). Written informed consent was signed by each patient.

**Study design**

A total of 208 patients with confirmed severe acute respiratory syndrome coronavirus 2 (SARS-CoV-2) infection were included. Sixteen patients with existing liver diseases, including liver cirrhosis, non-alcoholic fatty liver disease, alcoholic liver disease and chronic viral hepatitis, were excluded. The participants consist of 29 severe COVID-19 patients and 163 cases with mild symptoms. All patients were sorted into mild or severe group according to National Guidelines for 2019 novel coronavirus-associated pneumonia (the fifth revised version). The participants were divided into patients with (n=75) and without (n=117) liver injury group at admission. Furthermore, the individuals were sorted into patients with (n=133) and without liver injury group (n=59) during hospitalization. Patients with liver injury were defined as any parameter more than the upper limit unit (ULN) of normal value of ALT, AST and total bilirubin on admission. Univariate and multivariate logistic regression analyses were performed to identify the risk factors in COVID-19 patients with liver injury, and the study design was presented in **Figure S1**.

**Data collection**

The data including demographic data, clinical features, laboratory test results, treatment plans and therapeutic outcomes of 192 patients with COVID-19 was collected from hospital management system at different time points. All patients completed the tests on the first day after admission. The analyses of laboratory test results were completed every three days during hospitalization. All related tests such as routine blood test, procalcitonin (PCT) test, erythrocyte sedimentation rate (ESR) test, cytokine test (including TNF-α, IL-2, IL-4, IL-6, IL-10 and IL-17A), coagulation profile test, serum biochemical test (including renal and liver function, serum lipid) and T lymphocyte subsets analysis (CD3+, CD4+, CD8+ T-cell) were performed at the Department of Laboratory Medicine, Chongqing Public Health Center.

**Statistical analysis**

Data were analyzed using SPSS 22.0 (SPSS Inc., Chicago, IL, USA). Categorical data were presented as percentage of frequency and compared using χ^2^ or Fisher exact test. Continuous data were presented as medians with interquartile range (IQR) and compared using Student’s t-test or Mann-Whitney U test. Univariate and multivariable logistic regression analyses were used to identify the risk factors. P<0.05 was considered to indicate a statistically significant difference.


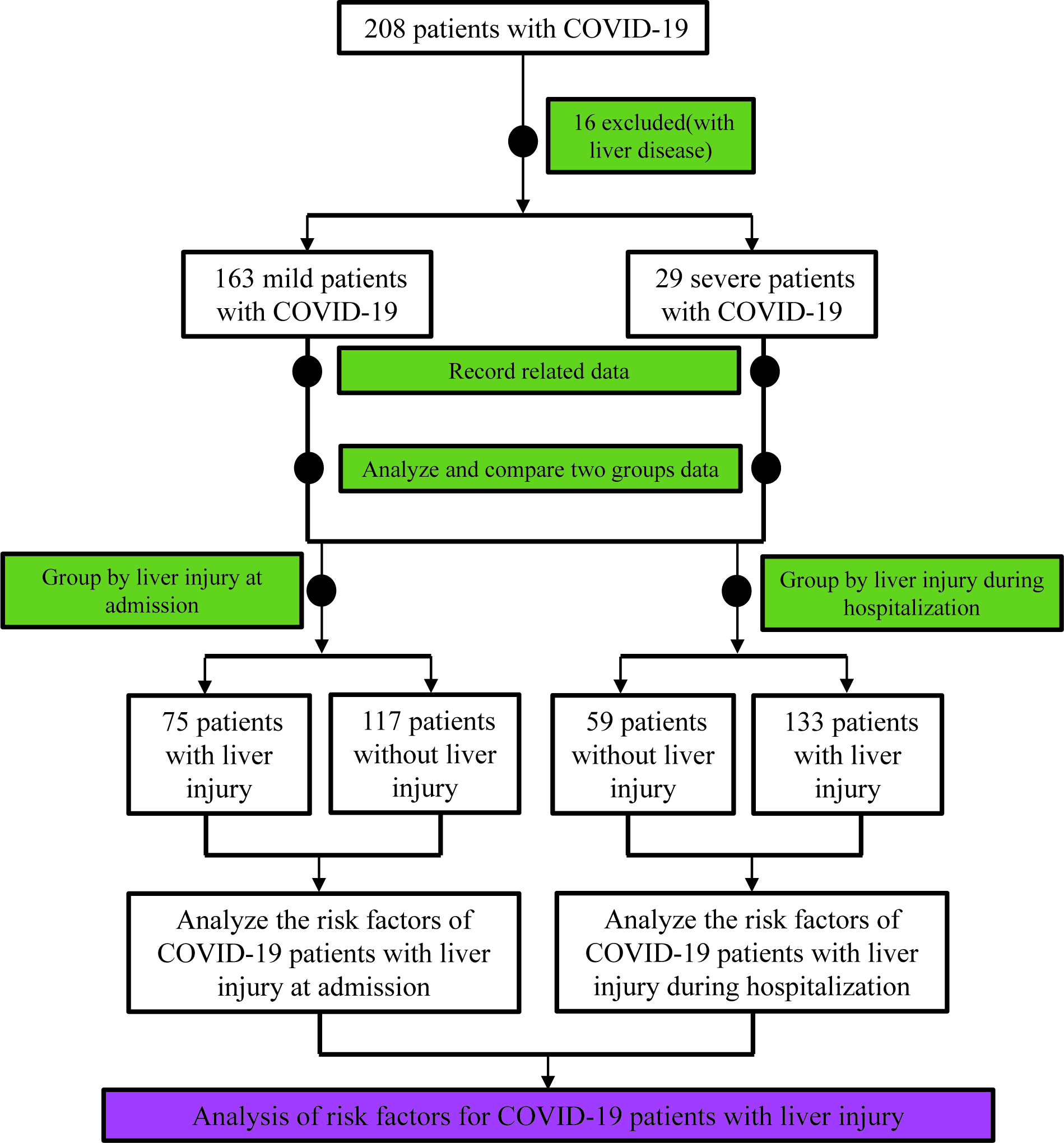


**Figure S1. Flowchart of study design.**


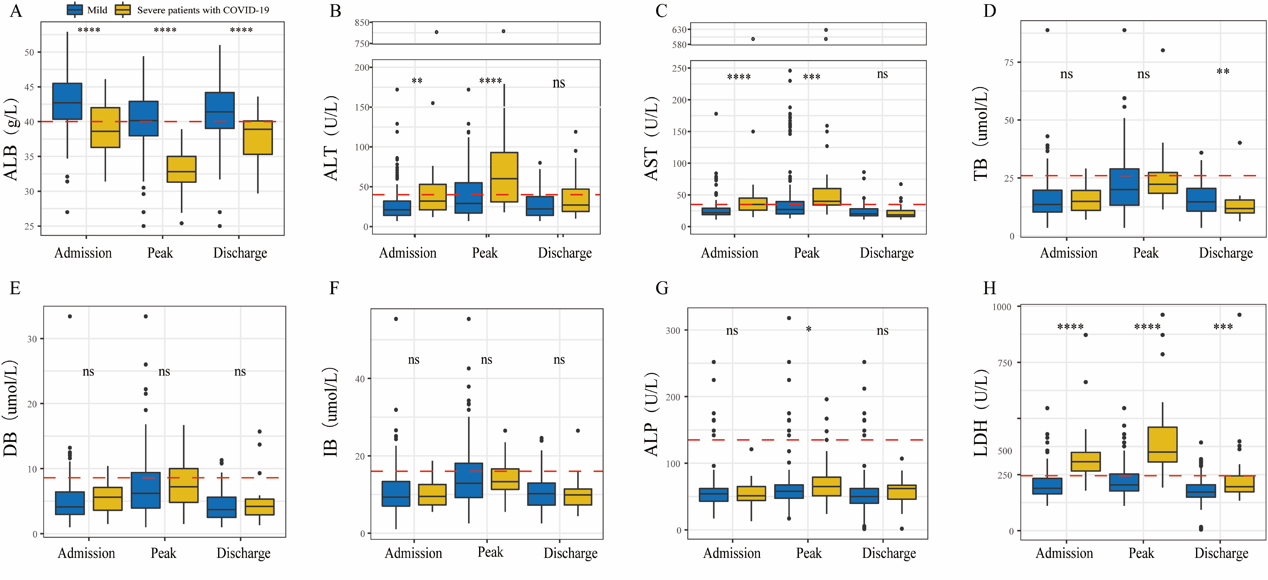


**Figure S2. Routine laboratory tests were performed on COVID-19 patients at admission, during hospitalization and at discharge.** (A) The levels of serum albumin were examined in severe patients compared to mild group. (B and C) The production of ALT and AST was evaluated in patients with severe and mild COVID-19. (D-F) The expression of TB, DB and IB was determined in COVID-19 patients. (G and H) The levels of ALP and LDH in recruited patients were assessed at different stages. ^*^P<0.05, ^**^P<0.01, ^***^P<0.001, ^****^P<0.0001. Peak indicates the highest value of each index during hospitalization.

**Table S1. Analysis of clinical features of COVID-19 patients enrolled in this study**

| Variable | All patients(n=192) | Mild patients with COVID-19 (n=163) | Severe patients with COVID-19 (n=29) | P-value |
| --- | --- | --- | --- | --- |
| Sex (Male/Female) | 98/94 | 81/82 | 17/12 | 0.494 |
| Age, years (mean (SD)) | 46.11 (16.48) | 44.25 (16.35) | 56.62 (13.10) | <0.001 |
| Hospitalization time (median [IQR]) | 17.00 [13.00, 23.00] | 16.00 [12.00, 21.00] | 24.00 [17.00, 34.00] | <0.001 |
| Coexisting disorder - no.(%) |  |  |  |  |
| Diabetes | 17 (8.9) | 7 (4.3) | 10 (34.5) | <0.001 |
| Hypertension | 23 (12.0) | 18 (11.0) | 5 (17.2) | 0.354 |
| Coronary heart disease | 5 (2.6) | 3 (1.8) | 2 (6.9) | 0.165 |
| Cancer | 8 (4.2) | 8 (4.9) | 0 (0.0) | 0.609 |
| Chronic obstructive pulmonary disease | 4 (2.1) | 4 (2.5) | 0 (0.0) | 1.000 |
| Chronic renal disease | 2 (1.0) | 2 (1.2) | 0 (0.0) | 1.000 |
| Liver injury - no.(%) | 75 (39.1) | 58 (35.6) | 17 (58.6) | 0.033 |
| Severity of liver injury - no. (%) |  |  |  | 0.415 |
| Mild | 66（88.0） | 52（89.7） | 14（82.4） |  |
| Severe | 9 (12.0) | 6 (10.3) | 3 (17.6) |  |
| Type of liver injury - no. (%) |  |  |  | 0.058 |
| Hepatocelluar | 34 (49.3) | 23 (44.2) | 11 (64.7) |  |
| Cholestatic | 24 (34.8) | 22 (42.3) | 2 (11.8) |  |
| Mixed | 11 (15.9) | 7 (13.5) | 4 (23.5) |  |
| Symptoms-no.(%) |  |  |  |  |
| Fever | 97 (50.5) | 75 (46.0) | 22 (75.9) | 0.006 |
| Fatigue | 41 (21.4) | 28 (17.2) | 13 (44.8) | 0.002 |
| Cough | 67 (34.9) | 60 (36.8) | 7 (24.1) | 0.211 |
| Sputum | 71 (37.0) | 56 (34.4) | 15 (51.7) | 0.095 |
| Muscle ache | 17 (8.9) | 16 (9.8) | 1 (3.4) | 0.477 |
| Shortness of breath | 41 (21.4) | 24 (14.7) | 17 (58.6) | <0.001 |
| Chest tightness | 28 (14.6) | 11 (6.7) | 17 (58.6) | <0.001 |
| Hemoptysis | 3 (1.6) | 2 (1.2) | 1 (3.4) | 0.39 |
| Dizziness | 21 (10.9) | 17 (10.4) | 4 (13.8) | 0.531 |
| Headache | 19 (9.9) | 15 (9.2) | 4 (13.8) | 0.497 |
| Runny | 12 (6.2) | 11 (6.7) | 1 (3.4) | 0.697 |
| Stuffy nose | 5 (2.6) | 4 (2.5) | 1 (3.4) | 0.563 |
| Sore throat | 15 (7.8) | 12 (7.4) | 3 (10.3) | 0.705 |
| Anorexia | 43 (22.4) | 22 (13.5) | 21 (72.4) | <0.001 |
| Nausea | 6 (3.1) | 5 (3.1) | 1 (3.4) | 1.000 |
| Vomiting | 1 (0.5) | 1 (0.6) | 0 (0.0) | 1.000 |
| Abdominal pain | 5 (2.6) | 5 (3.1) | 0 (0.0) | 1.000 |
| Diarrhea | 22 (11.5) | 18 (11.0) | 4 (13.8) | 0.751 |
| Laboratory findings - (median [IQR]) |  |  |  |  |
| White blood cell count, × 10^9^ per L | 5.05 [4.03, 6.38] | 5.05 [4.09, 6.36] | 5.31 [3.88, 6.68] | 0.849 |
| Neutrophil count, × 10^9^ per L | 2.99 [2.27, 4.12] | 2.97 [2.20, 3.96] | 3.30 [2.66, 4.74] | 0.092 |
| Lymphocyte count, × 10^9^per L | 1.35 [1.03, 1.89] | 1.44 [1.12, 1.93] | 0.87 [0.73, 1.13] | <0.001 |
| Platelet count, × 10^9^per L | 189.50 [139.50, 230.50] | 194.00 [142.00, 231.00] | 168.00 [126.00, 189.00] | 0.091 |
| Haemoglobin, g/L | 134.00 [124.75, 146.25] | 136.00 [124.50, 147.00] | 130.00 [125.00, 140.00] | 0.252 |
| Monocyte count, × 10^9^ per L | 0.39 [0.32, 0.50] | 0.40 [0.32, 0.50] | 0.33 [0.25, 0.50] | 0.157 |
| Red blood cell count-(mean (SD)), × 10^12^ per L | 4.42 (0.52) | 4.45 (0.53) | 4.23 (0.37) | 0.038 |
| Fecal occult blood-no.(%) | 5 (3.5) | 3 (2.3) | 2 (14.3) | 0.074 |
| Albumin, g/L | 42.08 (4.18) | 42.64 (3.97) | 38.92 (3.99) | <0.001 |
| Globulin, g/L | 26.05 [23.90, 29.92] | 26.00 [23.90, 29.80] | 27.70 [24.80, 30.30] | 0.283 |
| Total bile acid, umol/L | 3.55 [2.30, 6.20] | 3.50 [2.30, 6.00] | 4.40 [2.50, 8.00] | 0.236 |
| Triglyceride, mm/L | 1.53 [1.14, 2.09] | 1.49 [1.07, 2.02] | 1.78 [1.23, 3.60] | 0.093 |
| Total cholesterol , mm/L | 4.23 [3.69, 5.05] | 4.21 [3.67, 5.00] | 4.43 [3.93, 5.67] | 0.118 |
| High density lipoprotein, mm/L | 1.03 [0.88, 1.21] | 1.03 [0.87, 1.20] | 1.02 [0.92, 1.25] | 0.711 |
| Low-density lipoprotein, mm/L | 2.32 [2.03, 2.89] | 2.30 [1.99, 2.82] | 2.51 [2.14, 3.09] | 0.179 |
| Procalcitonin, ng/mL | 0.02 [0.02, 0.04] | 0.02 [0.02, 0.04] | 0.05 [0.02, 0.07] | <0.001 |
| C-reactive protein, mg/L | 5.13 [2.71, 22.38] | 4.09 [2.33, 13.04] | 35.41 [21.90, 59.16] | <0.001 |
| Erythrocyte sedimentation rate, mm/h | 33.00 [19.00, 54.00] | 30.00 [12.50, 45.00] | 55.50 [34.25, 81.00] | <0.001 |
| D-dimer, μg/mL | 0.20 [0.12, 0.31] | 0.18 [0.11, 0.30] | 0.24 [0.19, 0.45] | 0.015 |
| Fibrinogen-(mean (SD)), g/L | 4.05 (1.15) | 4.00 (1.17) | 4.33 (0.98) | 0.153 |
| Prothrombin time, s | 11.75 [11.20, 12.40] | 11.80 [11.20, 12.35] | 11.70 [11.20, 12.40] | 0.542 |
| Activated partial thromboplastin time, s | 39.20 [35.72, 42.92] | 39.00 [35.65, 42.40] | 40.00 [36.00, 45.00] | 0.202 |
| International normalized ratio | 0.95 [0.91, 0.99] | 0.96 [0.91, 0.99] | 0.94 [0.90, 0.97] | 0.17 |
| CD4^+^ T cell, per ul | 422.00 [264.00, 608.50] | 449.00 [313.75, 641.00] | 241.00 [161.00, 353.50] | <0.001 |
| CD8^+^ T cell, per ul | 305.50 [182.25, 463.50] | 320.50 [214.25, 491.25] | 157.00 [126.25, 269.00] | <0.001 |
| CD4^+^ T /CD8^+^ T cell | 1.40 [1.13, 1.99] | 1.41 [1.13, 2.00] | 1.40 [0.96, 1.89] | 0.344 |
| CD3^+^ T cell, per ul | 804.00 [530.75, 1119.75] | 846.00 [603.00, 1195.50] | 414.00 [318.75, 562.00] | <0.001 |
| γ-interferon, pg/mL | 1.44 [1.04, 2.56] | 1.12 [0.95, 1.98] | 1.44 [1.21, 2.58] | 0.514 |
| TNF-α, pg/mL | 1.18 [0.62, 1.64] | 1.05 [0.00, 1.64] | 1.31 [0.93, 1.64] | 0.281 |
| IL-10, pg/mL | 4.39 [2.21, 5.47] | 2.17 [1.45, 2.84] | 5.12 [4.39, 7.96] | <0.001 |
| IL-6, pg/mL | 4.24 [1.45, 14.29] | 2.95 [1.45, 6.59] | 13.94 [1.73, 28.59] | 0.037 |
| IL-4, pg/mL | 0.99 [0.42, 1.31] | 1.05 [0.13, 1.31] | 0.94 [0.68, 1.17] | 0.908 |
| IL-2, pg/mL | 1.05 [0.86, 1.55] | 0.95 [0.87, 1.29] | 1.15 [0.85, 1.55] | 0.833 |
| IL-17A, pg/mL | 14.23 [3.19, 20.77] | 5.95 [0.00, 11.80] | 18.80 [14.23, 23.87] | 0.017 |

**Table S2. The characteristics of COVID-19 patients with or without liver injury at admission**

| Variable | COVID-19 patients without liver injury at admission (n=117) | COVID-19 patients with liver injury at admission (n=75) | P-value |
| --- | --- | --- | --- |
| Sex (Male/Female) | 49/68 | 49/26 | 0.002 |
| Age,years (mean (SD)) | 47.36 (14.92) | 44.17 (18.60) | 0.192 |
| Hospitalization time (median [IQR]) | 17.00 [14.00, 23.00] | 17.00 [12.00, 21.50] | 0.366 |
| Severe patients with COVID-19 (%) | 12 (10.3) | 17 (22.7) | 0.023 |
| Coexisting disorder - no.(%) |  |  |  |
| Diabetes | 10 (8.5) | 7 (9.3) | 1.000 |
| Hypertension | 14 (12.0) | 9 (12.0) | 1.000 |
| Coronary heart disease | 4 (3.4) | 1 (1.3) | 0.650 |
| Cancer | 6 (5.1) | 2 (2.7) | 0.486 |
| Chronic obstructive pulmonary disease | 1 (0.9) | 3 (4.0) | 0.301 |
| Chronic renal disease | 2 (1.7) | 0 (0.0) | 0.521 |
| Symptoms-no.(%) |  |  |  |
| Fever | 61 (52.1) | 36 (48.0) | 0.681 |
| Fatigue | 27 (23.1) | 14 (18.7) | 0.589 |
| Cough | 41 (35.0) | 26 (34.7) | 1.000 |
| Sputum | 39 (33.3) | 32 (42.7) | 0.221 |
| Muscle ache | 13 (11.1) | 4 (5.3) | 0.201 |
| Shortness of breath | 25 (21.4) | 16 (21.3) | 1.000 |
| Chest tightness | 14 (12.0) | 14 (18.7) | 0.214 |
| Hemoptysis | 0 (0.0) | 3 (4.0) | 0.058 |
| Dizziness | 16 (13.7) | 5 (6.7) | 0.158 |
| Headache | 14 (12.0) | 5 (6.7) | 0.323 |
| Runny | 5 (4.3) | 7 (9.3) | 0.221 |
| Stuffy nose | 3 (2.6) | 2 (2.7) | 1.000 |
| Sore throat | 13 (11.1) | 2 (2.7) | 0.051 |
| Anorexia | 20 (17.1) | 23 (30.7) | 0.034 |
| Nausea | 3 (2.6) | 3 (4.0) | 0.680 |
| Vomiting | 0 (0.0) | 1 (1.3) | 0.391 |
| Abdominal pain | 5 (4.3) | 0 (0.0) | 0.159 |
| Diarrhea | 11 (9.4) | 11 (14.7) | 0.353 |
| Laboratory findings - (median [IQR]) |  |  |  |
| White blood cell count, × 10^9^ per L | 4.82 [3.94, 5.86] | 5.43 [4.35, 6.92] | 0.013 |
| Neutrophil count, × 10^9^ per L | 2.92 [2.13, 3.90] | 3.14 [2.50, 4.44] | 0.108 |
| Lymphocyte count, × 10^9^per L | 1.31 [1.03, 1.76] | 1.44 [0.94, 1.97] | 0.468 |
| Platelet count, × 10^9^per L | 185.00 [137.00, 218.00] | 198.00 [140.50, 247.50] | 0.172 |
| Haemoglobin, g/L | 133.00 [124.00, 144.00] | 138.00 [127.00, 148.50] | 0.083 |
| Monocyte count, × 109 per L | 0.40 [0.32, 0.51] | 0.39 [0.30, 0.46] | 0.655 |
| Red blood cell count-(mean (SD)), × 10^12^ per L | 4.38 (0.50) | 4.48 (0.55) | 0.199 |
| Fecal occult blood-no.(%) | 3 (3.4) | 2 (3.5) | 1.000 |
| Albumin, g/L | 42.06 (3.97) | 42.10 (4.51) | 0.95 |
| Globulin, g/L | 26.20 [23.80, 29.80] | 26.00 [24.65, 30.00] | 0.45 |
| Total bile acid, umol/L | 3.50 [2.30, 6.20] | 3.70 [2.60, 6.20] | 0.258 |
| Triglyceride, mm/L | 1.47 [1.04, 2.03] | 1.69 [1.22, 2.14] | 0.208 |
| Total cholesterol , mm/L | 4.05 [3.49, 5.00] | 4.28 [3.86, 5.07] | 0.179 |
| High density lipoprotein, mm/L | 1.01 [0.86, 1.13] | 1.05 [0.91, 1.25] | 0.257 |
| Low-density lipoprotein, mm/L | 2.24 [1.90, 2.79] | 2.43 [2.11, 2.93] | 0.204 |
| Procalcitonin, ng/mL | 0.02 [0.02, 0.04] | 0.02 [0.02, 0.06] | 0.886 |
| C-reactive protein, mg/L | 5.15 [2.83, 19.30] | 4.97 [2.09, 41.02] | 0.778 |
| Erythrocyte sedimentation rate, mm/h | 30.00 [12.75, 54.50] | 36.00 [27.00, 47.00] | 0.142 |
| D-dimer, μg/mL | 0.18 [0.12, 0.30] | 0.21 [0.12, 0.50] | 0.276 |
| Fibrinogen-(mean (SD)), g/L | 3.99 (1.03) | 4.15 (1.32) | 0.356 |
| Prothrombin time, s | 11.80 [11.30, 12.30] | 11.70 [10.95, 12.50] | 0.42 |
| Activated partial thromboplastin time, s | 39.20 [36.00, 43.10] | 39.20 [35.25, 42.25] | 0.718 |
| International normalized ratio | 0.96 [0.92, 0.99] | 0.94 [0.90, 0.99] | 0.178 |
| CD4^+^ T cell, per ul | 475.00 [316.00, 679.00] | 356.00 [196.00, 614.00] | 0.002 |
| CD8^+^ T cell, per ul | 316.00 [217.00, 426.00] | 290.00 [140.00, 490.00] | 0.211 |
| CD4^+^ T /CD8^+^ T cell | 1.44 [1.17, 2.00] | 1.32 [0.96, 1.77] | 0.065 |
| CD3^+^ T cell, per ul | 949.51 (449.71) | 785.96 (532.94) | 0.028 |
| γ-interferon, pg/mL | 1.44 [1.11, 2.04] | 2.55 [1.68, 3.31] | <0.001 |
| TNF-α, pg/mL | 1.26 [0.91, 1.83] | 2.12 [1.13, 3.43] | 0.002 |
| IL-10, pg/mL | 4.51 [2.52, 6.38] | 5.31 [4.15, 8.43] | 0.032 |
| IL-6, pg/mL | 8.21 [3.88, 18.43] | 10.01 [6.00, 24.50] | 0.117 |
| IL-4, pg/mL | 0.90 [0.62, 1.27] | 1.31 [0.78, 1.99] | 0.005 |
| IL-2, pg/mL | 1.05 [0.86, 1.31] | 1.45 [1.17, 2.48] | <0.001 |
| IL-17A, pg/mL | 11.80 [4.26, 18.45] | 18.80 [7.11, 27.61] | 0.007 |

**Table S3. Identification of novel risk factors in COVID-19 patients with liver injury at admission**

| Variable | Univariable OR (95% CI) | P-value | Multivariable OR (95% CI) | P-value |
| --- | --- | --- | --- | --- |
| Gender | 0.33 (0.15-0.74) | 0.008 | 0.31(0.11-0.80) | 0.007 |
| Anorexia | 1.83 (0.77-4.44) | 0.173 |  |  |
| White blood cell count, × 10^9^ per L | 1.18(0.99-1.46) | 0.088 |  |  |
| IL-10, pg/mL | 1.02(0.93-1.11) | 0.711 |  |  |
| IL-4, pg/mL | 2.32(1.4-4.19) | 0.002 |  |  |
| IL-2, pg/mL | 3.20(1.69-7.03) | 0.001 | 4.07(1.25-14.20) | <0.001 |
| IL-17A, pg/mL | 1.55(1.32-1.69) | 0.008 | 1.63(1.23-2.40) | 0.004 |
| γ-interferon, pg/mL | 1.04(0.97-1.19) | 0.34 |  |  |
| TNF-α, pg/mL | 1.73(1.27-2.49) | 0.001 |  |  |
| CD4^+^ T cell, per ul | 2.65(1.91-3.98) | <0.001 | 3.90(1.41-12.17) | <0.001 |
| CD8^+^ T cell, per ul | 1.01(0.98-1.04) | 0.599 |  |  |
| CD3^+^ T cell, per ul | 1.06(1.02-1.1) | 0.008 |  |  |

**Table S4. The features of COVID-19 patients with liver injury during hospitalization**

| Variable | COVID-19 patients without liver injury during hospitalization (n=59) | COVID-19 patients with liver injury during hospitalization (n=133) | P-value |
| --- | --- | --- | --- |
| Gender (Male/Female) | 23/36 | 75/58 | 0.029 |
| Age, years (mean (SD)) | 46.44 (15.13) | 45.97 (17.10) | 0.856 |
| Hospitalization time (median [IQR]) | 16.00 [12.00, 20.00] | 17.00 [13.00, 24.00] | 0.306 |
| Severe patients with COVID-19 (%) | 4 (6.8) | 25 (18.8) | 0.047 |
| Coexisting disorder - no.(%) |  |  |  |
| Diabetes | 4 (6.8) | 13 (9.8) | 0.592 |
| Hypertension | 4 (6.8) | 19 (14.3) | 0.157 |
| Coronary heart disease | 1 (1.7) | 4 (3.0) | 1.000 |
| Cancer | 1 (1.7) | 7 (5.3) | 0.439 |
| Chronic obstructive pulmonary disease | 0 (0.0) | 4 (3.0) | 0.314 |
| Chronic renal disease | 0 (0.0) | 2 (1.5) | 1.000 |
| Symptoms-no.(%) |  |  |  |
| Fever | 33 (55.9) | 74 (55.6) | 1.000 |
| Fatigue | 14 (23.7) | 30 (22.6) | 0.854 |
| Cough | 27 (45.8) | 56 (42.1) | 0.640 |
| Sputum | 24 (40.7) | 58 (43.6) | 0.753 |
| Muscle ache | 4 (6.8) | 14 (10.5) | 0.593 |
| Shortness of breath | 15 (25.4) | 32 (24.1) | 0.857 |
| Chest tightness | 7 (11.9) | 27 (20.3) | 0.219 |
| Hemoptysis | 1 (1.7) | 6 (4.5) | 0.441 |
| Dizziness | 11 (18.6) | 12 (9.0) | 0.089 |
| Headache | 7 (11.9) | 17 (12.8) | 1.000 |
| Runny | 3 (5.1) | 11 (8.3) | 0.556 |
| Stuffy nose | 1 (1.7) | 4 (3.0) | 1.000 |
| Sore throat | 7 (11.9) | 12 (9.0) | 0.603 |
| Anorexia | 13 (22.0) | 47 (35.3) | 0.091 |
| Nausea | 2 (3.4) | 20 (15.0) | 0.025 |
| Vomiting | 5 (8.5) | 12 (9.0) | 1.000 |
| Abdominal pain | 4 (6.8) | 4 (3.0) | 0.253 |
| Diarrhea | 29 (49.2) | 63 (47.4) | 0.876 |
| Drug used - no.(%) |  |  |  |
| Ritonavir | 38 (64.4) | 112 (84.2) | 0.002 |
| Prezista | 8 (13.6) | 17 (12.8) | 1.000 |
| Arbidol | 15 (25.4) | 35 (26.3) | 1.000 |
| Ribavirin | 15 (25.4) | 42 (31.6) | 0.494 |
| Chloroquine | 7 (11.9) | 26 (19.5) | 0.220 |
| Interferon | 55 (93.2) | 131 (98.5) | 0.073 |
| Antibiotics | 6 (10.2) | 34 (25.6) | 0.020 |
| Laboratory findings - (median [IQR]) |  |  |  |
| White blood cell count, × 10^9^ per L | 5.58 [4.79, 6.95] | 6.40 [4.84, 8.56] | 0.058 |
| Neutrophil count, × 10^9^ per L | 3.55 [2.75, 4.91] | 3.97 [2.89, 6.16] | 0.150 |
| Lymphocyte count, × 10^9^per L | 1.62 [1.25, 2.08] | 1.64 [1.27, 2.12] | 0.720 |
| Platelet count, × 10^9^per L | 223.00 [175.50, 281.50] | 252.00 [201.00, 339.00] | 0.044 |
| Haemoglobin, g/L | 131.00 [121.50, 143.50] | 137.00 [127.00, 150.00] | 0.023 |
| Monocyte count, × 10^9^ per L | 0.46 [0.36, 0.56] | 0.50 [0.39, 0.70] | 0.041 |
| Red blood cell count, × 10^12^ per L | 4.40 [4.09, 4.79] | 4.47 [4.16, 4.87] | 0.516 |
| Procalcitonin, ng/mL | 0.02 [0.02, 0.04] | 0.03 [0.02, 0.07] | 0.058 |
| C-reactive protein, mg/L | 7.61 [3.75, 22.08] | 11.56 [2.88, 46.46] | 0.750 |
| D-dimer, μg/mL | 0.18 [0.12, 0.31] | 0.24 [0.13, 0.68] | 0.062 |
| Fibrinogen, g/L | 4.33 [3.33, 4.81] | 4.37 [3.36, 5.25] | 0.327 |
| Prothrombin time, s | 11.80 [11.20, 12.40] | 12.00 [11.30, 12.50] | 0.232 |
| Activated partial thromboplastin time, s | 40.40 [37.40, 43.50] | 40.15 [36.30, 43.90] | 0.922 |
| International normalized ratio | 0.96 [0.92, 1.00] | 0.97 [0.92, 1.00] | 0.994 |
| CD4^+^ T cell, per ul | 600.00 [415.00, 925.75] | 366.00 [239.00, 574.00] | <0.001 |
| CD8^+^ T cell, per ul | 341.00 [265.25, 450.75] | 296.00 [146.00, 421.00] | 0.022 |
| CD4^+^ T /CD8^+^ T cell | 1.48 [1.26, 2.37] | 1.37 [1.08, 1.87] | 0.014 |
| CD3^+^ T cell, per ul | 1104.00 [758.50, 1435.00] | 708.00 [434.00, 1060.00] | <0.001 |
| γ-interferon, pg/mL | 1.44 [0.95, 2.08] | 1.72 [1.34, 2.75] | 0.057 |
| TNF-α, pg/mL | 0.86 [0.44, 2.05] | 1.58 [0.95, 2.70] | 0.014 |
| IL-10, pg/mL | 3.28 [1.72, 4.11] | 5.40 [3.79, 7.64] | <0.001 |
| IL-6, pg/mL | 1.72 [1.12, 6.24] | 11.21 [4.56, 27.58] | <0.001 |
| IL-4, pg/mL | 0.78 [0.41, 1.31] | 1.10 [0.65, 1.84] | 0.109 |
| IL-2, pg/mL | 1.10 [0.62, 1.40] | 1.19 [0.94, 1.77] | 0.035 |
| IL-17A, pg/mL | 6.73 [0.00, 20.95] | 14.57 [5.95, 20.77] | 0.092 |
